# Disulfiram, a drug widely used to control alcoholism, suppresses self-renewal of glioblastoma and overrides resistance to temozolomide

**DOI:** 10.18632/oncotarget.604

**Published:** 2012-10-08

**Authors:** Joanna Triscott, Cathy Lee, Kaiji Hu, Abbas Fotovati, Rachel Berns, Mary Pambid, Margaret Luk, Richard E. Kast, Esther Kong, Eric Toyota, Stephen Yip, Brian Toyota, Sandra E. Dunn

**Affiliations:** ^1^ Department of Pediatrics, University of British Columbia, Vancouver, BC; ^2^ Department of Pathology, Vancouver General Hospital, Vancouver, BC; ^3^ Department of Psychiatry, University of Vermont, Burlington, VT, USA; ^4^ Department of Pathology & Laboratory Medicine, Centre for Translational and Applied Genomics, BC Cancer Agency, Vancouver, BC; ^5^ Department of Surgery, British Columbia Cancer Agency, Vancouver, BC

**Keywords:** glioblastoma, disulfiram, temozolomide, brain tumor, drug resistance

## Abstract

Glioblastomas (GBM) are associated with high rates of relapse. These brain tumors are often resistant to chemotherapies like temozolomide (TMZ) and there are very few treatment options available to patients. We recently reported that polo-like kinase-1 (PLK1) is associated with the proliferative subtype of GBM; which has the worst prognosis. In this study, we addressed the potential of repurposing disulfiram (DSF), a drug widely used to control alcoholism for the past six decades. DSF has good safety profiles and penetrates the blood-brain barrier. Here we report that DSF inhibited the growth of TMZ resistant GBM cells, (IC90=100 nM), but did not affect normal human astrocytes. At similar DSF concentrations, self-renewal was blocked by ~100% using neurosphere growth assays. Likewise the drug completely inhibited the self-renewal of the BT74 and GBM4 primary cell lines. Additionally, DSF suppressed growth and self-renewal of primary cells from two GBM tumors. These cells were resistant to TMZ, had unmethylated MGMT, and expressed high levels of PLK1. Consistent with its role in suppressing GBM growth, DSF inhibited the expression of PLK1 in GBM cells. Likewise, PLK1 inhibition with siRNA, or small molecules (BI-2536 or BI-6727) blocked growth of TMZ resistant cells. Our studies suggest that DSF has the potential to be repurposed for treatment of refractory GBM.

## INTRODUCTION

Glioblastoma (GBM) is the most aggressive type of brain tumor with limited treatment options. The location and infiltrative nature of GBM tumors makes surgical resection and radiation frequently ineffective, thus recurrence is especially common. Under the current treatment regime of temozolomide (TMZ) and radiation the median expected survival following resection is only 14 months [1]. Resistance to the alkylating agent TMZ is common in GBM. Tumors expressing O6-methylguanine methyltransferase (MGMT) avoid growth inhibition by enzymatically removing the methyl groups added to DNA by TMZ [2, 3], however, even MGMT silenced cases acquire TMZ resistance [4]. For example, chronic exposure to TMZ has been shown to generate mutations in mismatch repair genes and offers an additional route of treatment resistance [5]. There are few options available to overcome GBM growth and recurrence.

Tumor re-growth and relapse is a major problem in treating GBM. A growing body of genetic analysis of GBM suggests that cell cycle and regulatory factors are key drivers of the disease that dictate patient survival [6-8]. Recently it has become apparent that GBM is comprised of a heterogeneous mixture of cells that have different properties that contribute to treatment resistance in animal models [9-14]. Most current treatments target the proliferative capacity of cancer cells, however, the ability of more undifferentiated populations of GBM cells to self-renew is often unaffected by chemotherapy [15, 16]. Self-renewal is a process that is controlled by cell cycle and allows the indefinite perpetuation of cells that are uncommitted to terminal tissue-specific lineages [13, 17-19]. A number of groups have successfully isolated and characterized GBM cell lines using culture conditions that retain the self-renewal properties of the primary tumor [11, 12, 20-22]. Propagation and *in vitro* assessment of brain tumor cell self-renewal is done using neurosphere tissue culture conditions [11, 23, 24]. The self-renewing properties of cells allow them to be serial passaged using these growth conditions and continually form new spheroid cell clusters. With the potential to evade current treatment protocols, there must be alternative methods developed that target both the cancer cell proliferation and self-renewal in order to prevent GBM relapse [9].

Polo-like kinase 1 (PLK1) is a key serine/threonine kinase involved in many essential cell cycle functions, such as: mitotic entry, centrosome maturation, cell cycle progression and cytokinesis [25-29]. Our group has demonstrated PLK1 to be a promising therapeutic target for brain tumors as it is highly over-expressed in cancer compared to normal tissue [30, 31]. As well, patients with GBM tumors expressing high levels of PLK1 have a greater probability of morbidity (or poorer prognosis) [30]. Recently we have shown that PLK1 inhibition delayed tumor growth in an orthotopic brain tumor model and also demonstrated PLK1 to be essential for sustaining the growth of tumorspheres [30]. Although chemical inhibitors of PLK1 are being developed for clinical use [32, 33], the long expensive process of drug development prompts the question of whether currently approved off-patent drugs may have undiscovered anti-cancer potential.

Disulfiram (DSF) has been safely used for the treatment of alcohol abuse for over sixty years. This compound is an inhibitor of the aldehyde dehydrogenase (ALDH) enzyme family, which is involved in the metabolism of alcohol, and has been suggested as a potential marker for self-renewing tumor cell populations [34, 35]. Although best characterized for its activity against ALDH, DSF is not a specific inhibitor and there is growing support that uncovers alternative effects of DSF on cell activity [36, 37]. Originally we identified DSF in a screen for drugs that inhibit tumor-initiating cells using the Prestwick Library (unpublished data). DSF was attractive to us because it is a small molecule and, as such, it crosses the blood-brain barrier [38-40]. In a position paper by Kast et al, DSF was proposed for the treatment of GBM [41]; therefore, we hypothesize that DSF will target drug resistant cells. This study provides in vitro evidence that DSF is an effective treatment for GBM and suggests it augments cytotoxicity of the currently used chemotherapeutic agent, TMZ. The data presented here proposes a new use for the clinically safe compound, DSF, as an alternative treatment for cancer patients.

## RESULTS

**Figure 1:**
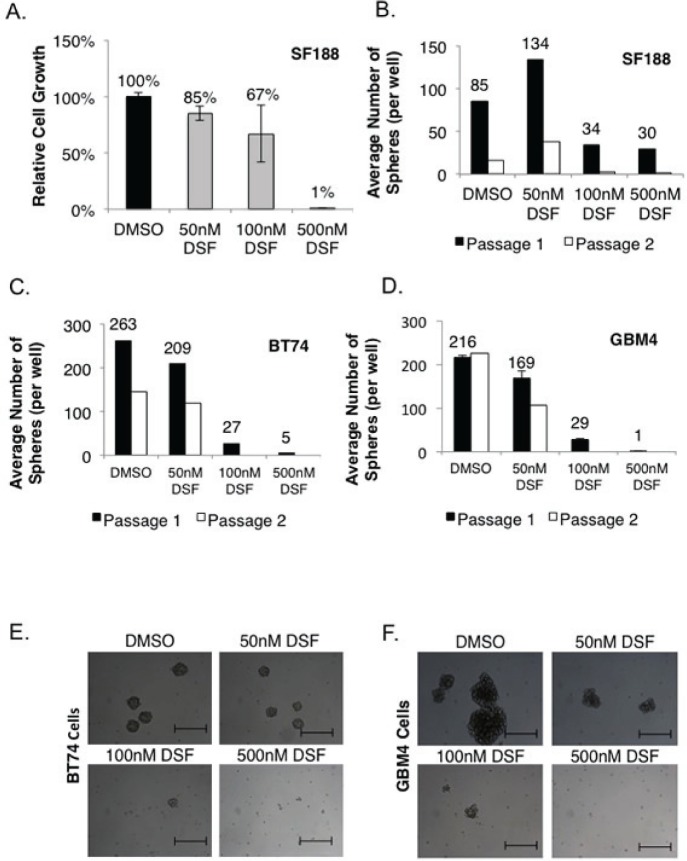
DSF inhibits GBM cell growth and self-renewal (A-B) SF188 cells were treated with 50, 100 or 500 nM DSF and tumor growth was assessed in monolayer or in serial neurosphere assays. (C-D) Adult GBM BT74 and GBM4 cells were treated with 50-500 nM DSF and self-renewal was assessed in neurosphere assays. Microscopy that demonstrates the effect of DSF treatment on BT74 (E) and GBM4 (F) neurosphere growth. Scale bar = 200 um

SF188 cells are pediatric GBM cells that are unaffected by TMZ at physiologically achievable concentrations (5-15 uM) based on cell growth assays (Supplemental Fig 1). These classically TMZ resistant cells were sensitive to 500 nM DSF, a sufficient concentration to suppress growth in monolayer by ~100% over 72 hours (Figure 1A), and the ability of these cells to self-renew was also completely inhibited (Figure 1B). BT74 cells are primary adult GBM cells, which are also refractory to TMZ [21, 42], however, they are sensitive to DSF in neurosphere self-renewal assays (Figure 1C). Likewise, GBM4 cells are sensitive to DSF in self-renewal assays (Figure 1D) and examples of the impact on BT74 and GBM4 neurosphere formation are illustrated in Figures 1E-F. Next we asked whether the combination of TMZ and DSF would have an additive cytotoxic effect. Low doses of 50 nM DSF or 10 uM TMZ had no effect as single agents, however together they inhibited proliferation and self-renewal by ~50% (Supplemental Figure 2A-B).

**Figure 2:**
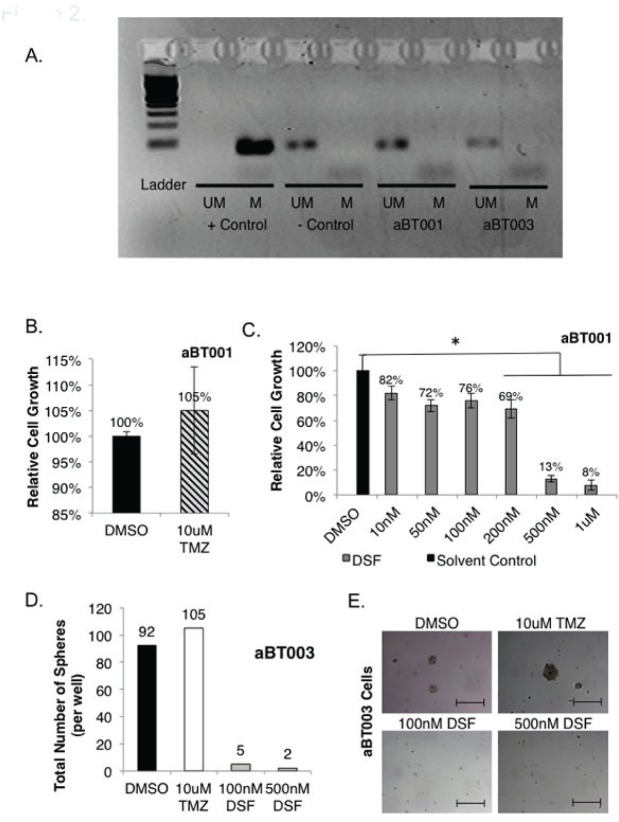
Freshly isolated GBM cells are sensitive to DSF yet resistant to TMZ (A) Primary GBM cells referred to aBT001 and aBT003 were isolated from adult patients with GBM. DNA was isolated from the tumors and subjected to MGMT analysis by PCR. In both cases the MGMT promoter was not methylated indicating that the protein would be expressed (M = methylated, UM = unmethylated). (B-C) The growth of aBT001 was unaffected by TMZ however DSF suppressed their growth by as much as 92% with a single treatment. Cell growth was assessed after 72 hrs. D-E) TMZ was ineffective at suppressing self-renewal when aBT003 cells were exposed to the drug. However DSF suppressed self-renewal by 95-98% based on a single exposure. Scale bar = 200 um.

Freshly isolated GBM cells were obtained from two adult patients, and denoted aBT001 and aBT003, respectively. GBM that have mutations in IDH1 or IDH2 are reported to undergo metabolic remodeling that influences the tumor survival program in response to treatment and hypoxia [43, 44]. Sequencing of IDH1 and IDH2 was negative for mutation in either tumor sample (data not shown). Both cases had unmethylated MGMT (Figure 2A) suggesting that they may be refractory to TMZ. As expected, TMZ did not inhibit the growth of aBT001 in monolayer (Figure 2B). However, these cells were sensitive to DSF, where 500 nM inhibited growth by 87% after 72 hours (Figure 2C). The aBT003 cells were also refractory to TMZ in neurosphere assays (Figure 2D). Conversely, DSF inhibited cell self-renewal capacity by 95-98% (Figure 2D-E). It is noteworthy that while DSF inhibited the growth of GBM cells it had no effect on the proliferation of normal human astrocytes at concentrations up to 10 uM, (Supplemental Figure 3).

**Figure 3:**
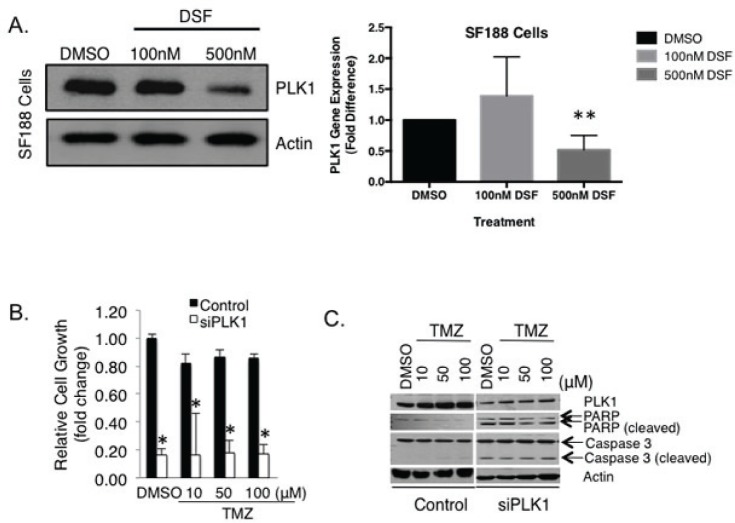
DSF inhibits the expression of PLK1 in pediatric GBM SF188 cells (A) SF188 cells were exposed to DSF for 24 hrs using DMSO as a solvent control. Protein and transcript levels of PLK1 were assessed using immunoblotting or qRT-PCR. (B-C) PLK1 inhibition with siRNA inhibits SF188 cell growth and induced apoptosis based on PARP and caspase 3 cleavage. Scramble RNA oligo was transfected as a control. The efficacy of PLK1 inhibition on SF188 growth is exemplified in combination with 10 uM TMZ.

**Figure 4:**
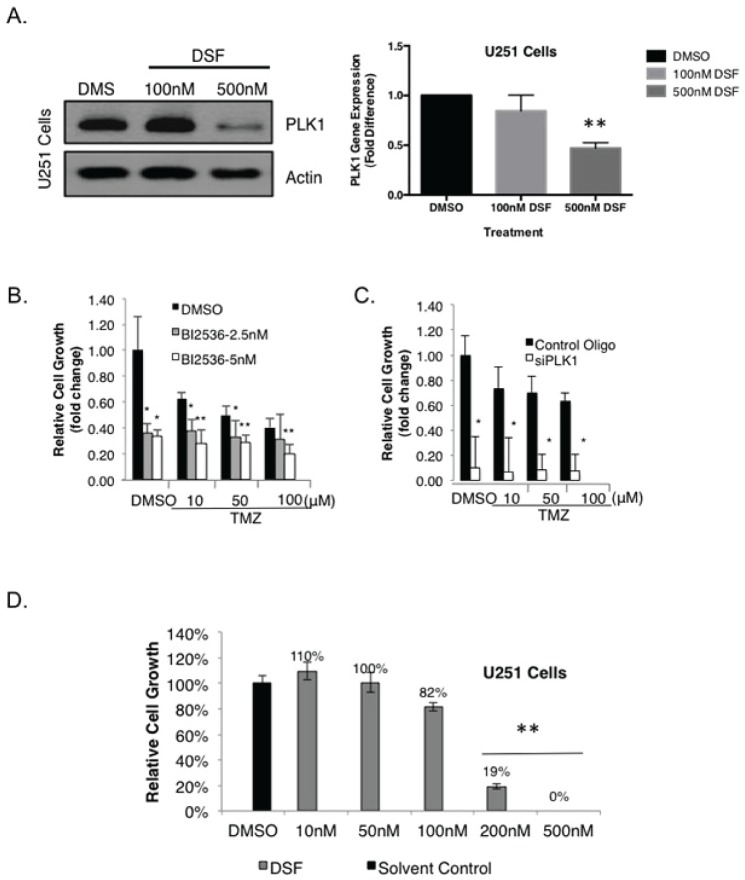
DSF inhibits the expression of PLK1 in adult GBM U251 cells (A) U251 cells were exposed to DSF for 24 hrs using DMSO as a solvent control. Protein and transcript levels of PLK1 were assessed using immunoblotting or qRT-PCR. (B-C) Inhibiting PLK1 with BI-2536 or siRNA inhibits their growth. The efficacy of PLK1 inhibition on U251 growth is exemplified in combination with 10 uM TMZ. (D) Likewise, DSF inhibits the growth of U251 cells in a dose-dependent manner using DMSO as a solvent control.

We recently reported that highly proliferative GBM express PLK1 and that these cells depend on it for survival [30], yet its role in the context of TMZ resistance has not been addressed. Because DSF had such a dramatic effect on the growth of GBM we questioned the mechanism and assessed the impact on PLK1. Notably, 500 nM DSF treatment for 24 hours inhibited PLK1 expression in SF188 cells (Figure 3A), as did 250 nM DSF (Supplemental Figure 4A). Inhibiting PLK1 with siRNA blocked the growth of these cells and induced apoptosis (Figure 3B-C). Similar results were also observed in U251 adult GBM cells as 500 nM DSF inhibited both PLK1 protein and transcript expression (Figure 4A). Lower doses of 250 nM DSF also inhibited PLK1 protein levels in the U251 cells (Supplemental Figure 4B). Inhibiting PLK1 with the small molecule BI-2536 or with siRNA blocked the U251 cell growth (Figure 4B-C). In addition, U251 cells treated with 200 nM DSF were suppressed in growth by 80% and 500 nM doses completely eliminated the cells (Figure 4D).

**Figure 5:**
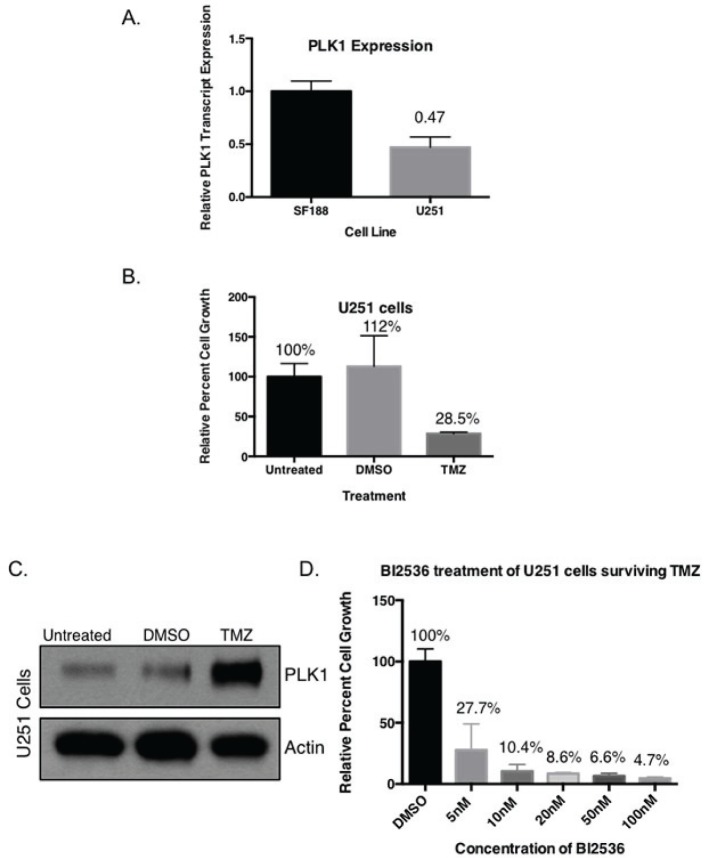
Targeting PLK1 inhibits growth of drug resistant cells with upregulated PLK1 protein (A) U251 cells have lower PLK1 transcript expression than TMZ resistant SF188 cells. U251 cells were treated with 10 uM TMZ every 2 days for a total of 7 days and (B) partial TMZ sensitivity is demonstrated in a growth assay. (C) Immunoblot demonstrating an increase in PLK1 protein levels in TMZ treated U251 cells compared to untreated and DMSO after 24 hours. Actin is used as a loading control protein. (D) The surviving TMZ resistant cells were re-plated and treated with increasing concentrations of BI-2536 for 5 days.

Previous published results by our group show SF188, BT74, BT241 and GBM4 all have between 203-470 fold overexpression of PLK1 transcript compared to normal human astrocytes [30]. In the present study, it was interesting to find the TMZ resistant SF188 cells have approximately double the amount of PLK1 transcript compared to the partially TMZ sensitive U251 cells (Figure 5A). We notice that while the U251 cells are reportedly sensitive to TMZ there was always ~30% of the cells that remained following treatment. For example, when we treated U251 cells with TMZ for 7 days about 70% of the cells died off leaving behind a residual population (Figure 5B). This population was harvested and evaluated for PLK1. Notably the PLK1 was much higher in the residual population compared to either the untreated control or the DMSO control (Figure 5C). Considering these results the study was repeated and the residual population that remained after treating the cells with TMZ for 7 day was then exposed to BI-2536. The TMZ resistant population was highly sensitive to PLK1 inhibition (Figure 5D). For example, 10 nM BI-2536 killed off 90% of the residual population. These findings suggest PLK1 to be a potential driver of TMZ resistance that can be overcome through therapeutic intervention.

**Figure 6:**
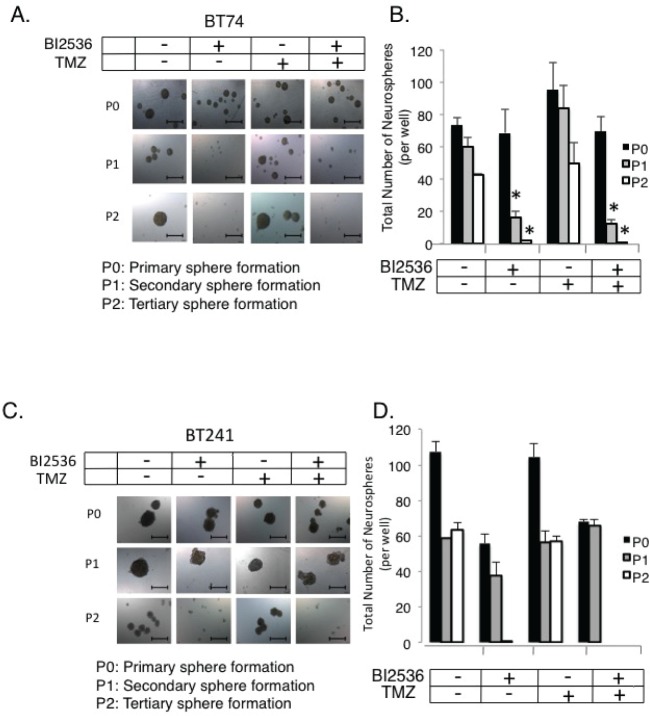
PLK1 inhibitors can be used to over-come TMZ resistance (A) BT74 cells are resistant to TMZ yet sensitive to PLK1 inhibition with BI-2536. (B) BT241 cells are a second example to which the cells are TMZ resistant yet sensitive to PLK1 inhibition. Both models are maintained as primary isolates and only cultured as neurospheres. The combination of TMZ and BI-2536 did not further improve self-renewal inhibition. Scale bar = 500 um.

**Figure 7:**
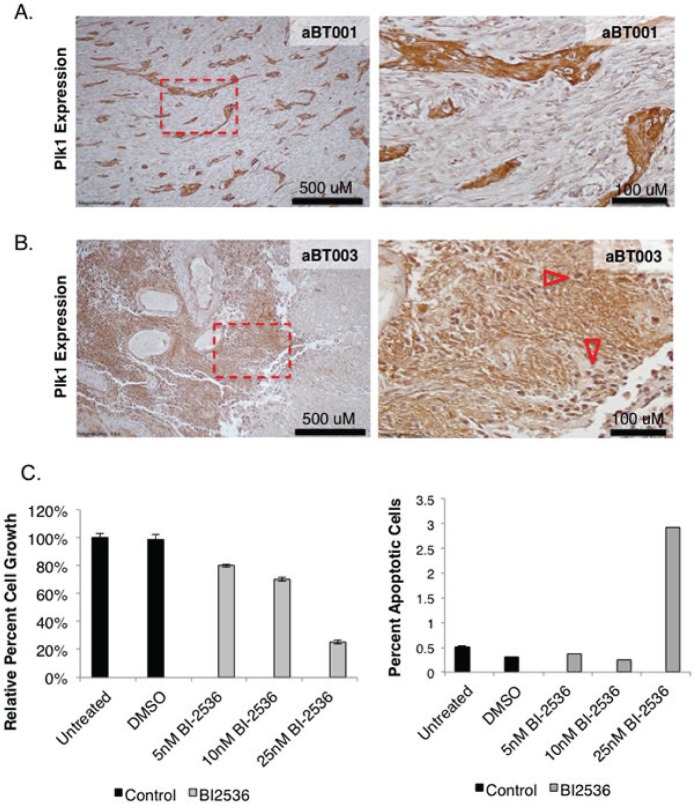
aBT001 and aBT003 express high levels of PLK1 (A) PLK1 levels were assessed in aBT001 and aBT003 by immunostaining. Both cases express high levels of PLK1. (B-C) The PLK1 inhibitor BI-2536 suppressed the growth of aBT001 and induced cell death.

The TMZ resistant BT74 cells [42] were also sensitive to PLK1 inhibition (Figure 6A-B). BT241 cells were also sensitive to PLK1 inhibition but not to TMZ treatment (Figure 6C-D). There was no additional benefit from combining BI-2536 and TMZ (Figure 6A-D). As previously mentioned freshly isolated aBT001 and aBT003 GBM cells were sensitive to DSF therefore we addressed whether they also expressed PLK1, which they did (Figure 7A-B). Given that the PLK1 target was expressed, aBT001 cells were treated for 72 hours with increasing amounts of BI-2536 that inhibited their growth by up to 80% and induced apoptosis (Figure 7C). Thus, PLK1 inhibition phenocopied the effect of DSF in blocking the growth of refractory GBM cells.

## DISCUSSION

Many challenges exist in the treatment of GBM, one of which is the immense problem of TMZ resistance. In some patients, the invasive tumor cells respond initially but most patients eventually relapse. For other patients, their tumors are resistant from the start of treatment. TMZ treatment is associated with low long-term survival rate and is ineffective at targeting cancer cell self-renewal that potentially repopulates the tumor [1, 45, 46]. In the present study, we demonstrate the efficacy of DSF in completely suppressing GBM cell growth in vitro. More importantly, we observed the same degree of inhibition in self-renewal assays. Concentrations as low as 100nM DSF suppressed cell growth in monolayer and this corresponded to an inhibition of self-renewal using neurosphere assays. Importantly, DSF was highly effective in cells that are refractory to TMZ.

Of notable importance, we showed that DSF was highly effective in situations where cells have developed TMZ resistance. Within this study we note that the treatment of cells with DSF results in downregulation of the important cell cycle kinase, PLK1. We have shown that TMZ treatment induces expression of PLK1 protein and speculate this phenomenon may have a role in the aggressive nature of recurrent GBM. It is interesting to note that TMZ resistant SF188 cells were found to have double the PLK1 transcript expression as partially TMZ sensitive U251 cells. Therefore, DSF has very promising potential for treatment of brain tumor patients. The combination of TMZ and DSF was helpful in some instances but not others. Several other studies showed that DSF was added to standard cancer chemotherapy agents: paclitaxel [47], gemcitabine [48], docetaxel [49], cyclophosphamide [50], and 5-fluorouracil [51]. This data also agrees with an in vitro study considering DSF toxicity to breast cancer stem cells [47]. Similarly, we show enhanced cytotoxicity to TMZ and investigate the use of DSF to target self-renewing primary cells.

DSF is being evaluated for other malignancies including an ongoing phase I clinical trial investigating the use of DSF to treat malignancies that have metastasized to the liver (ClinicalTrials.gov Identifier: NCT00742911). Another phase II trial is evaluating its use in combination with cisplatin for treating metastatic small cell lung carcinoma (ClinicalTrials.gov Identifier: NCT00312819). Verma et al. (1990) has also conducted phase II clinical trials of DSF as a method to decrease nephrotoxicity of cisplatin in a randomized study of cisplatin sensitive malignancies [52]. They reported no reduction in toxicity by combining the two drugs, however, this study had a markedly high patient drop- out rate making the data analysis inconsistent between groups [52]. It may be speculated that this study had issues due to the use of extremely high concentrations of greater than 3200 mg DSF. This level far exceeds the minimal concentrations required to elicit a response as suggested by our in vitro data, and is dangerously higher than the dose of 250mg/day used to treat alcoholism [52, 53]. At this point there has been no reported clinical experimentation in the use of DSF for the treatment of solid brain tumors with DSF only being used in clinical trials for metastatic cancers. We believe that DSF may have great benefit if used to treat primary tumors for a number of reasons. Not only is DSF an inexpensive and easily administered drug, it is able to cross the blood-brain barrier, which is a major limitation in brain therapeutic design [39, 40, 54].

In the context of this study, the mechanism of action for DSF was somewhat elusive for us. Other researchers speculate that metal chelating properties of DSF metabolites cause the initiation of apoptosis through redox induced oxidative stress [55]. Another theory is that these metal chelating properties cause the inhibition of the proteasome; a multisubunit complex involved with protein degradation pathways [56]. Studies in breast cancer suggest that DSF may modulate the NFκB pathway, which is a very often inappropriately activated in GBM, and is regulated in a proteasome dependent manner [47, 57]. These studies were conducted using the combination of DSF with the metal ions (eg. CuCl2), however, here we demonstrate exceptional activity of DSF in the absence of metal supplementation and question an alternative mechanism of action for DSF on cancer cells.

We initially suspected that DSF killed cells through ALDH inhibition [34]; however, this was not the case. We noted that while DSF inhibits ALDH activity it was at higher doses of the drug then required to suppress the growth of GBM cells. Additionally, blocking ALDH activity with a pan inhibitor (DEAB) did not suppress growth to the degree of DSF. ALDH1A1 and ALDH1A3 isoforms were also individually silenced with siRNA however this resulted in little or no growth suppression (data not shown) and loss of ALDH did not induce cell death in GBM cells. These studies redirected our focus toward a pathway that may cause considerable cell death and to this end we address the possible link with PLK1. We knew that GBM cells were absolutely dependent upon PLK1 as we recently reported that inhibiting expression suppressed the growth of GBM cells by as much as 100% and this was associated with cell death [30]. Serendipitously we noted that DSF also inhibited PLK1 expression in both SF188 and U251 cells. This novel mechanism would have broad reaching implications given that PLK1 is central to the growth of many types of cancer [30, 58-60]. This is the first demonstration of an off-patent drug that inhibits its expression and as such it opens up several new lines of investigation.

In the present study we demonstrated the efficacy of DSF in suppressing refractory GBM growth and self-renewal at low concentrations. Coupled with these findings, DSF has been used safely in humans for over half a century and therefore we believe it has excellent potential to be repositioned for the treatment of GBM.

## METHODS

### Cell culture

The American Tissue Culture Collection (ATCC) supplied pediatric GBM SF188 (TMZ resistant), adult GBM U251 (partial TMZ sensitivity), and normal human astrocyte cells. GBM4 and BT74 GBM cells are well-established GBM models that were obtained from Wakimoto et al. [20, 21, 42, 61] (BT74 cells originally isolated and characterized by James CD et al, and denoted as GBM6 [21]). BT241 is another primary isolate model that was isolated from a patient with GBM, and obtained from Singh et al [11, 62]. GBM4, BT74 and BT241 cells have all been previously characterized both in vitro and in vivo using xenograft experiments [11, 20-22, 62]. Primary patient isolated cells, aBT001 and aBT003, were isolated using methods previously described [22, 63]. All primary samples were acquired in accordance with the guidelines of the Institutional Review Board, and along with patient consent. SF188, U251, and aBT001 cells were grown in monolayer using Minimum Essential Medium/Earle's Balanced Salt Solutions (MEM/EBSS) [Hyclone, Logan UT, USA] and Dulbecco's Modified Eagle Medium (DMEM)/High Glucose (Hyclone), respectively, supplemented with 10% fetal bovine serum (FBS). BT74, aBT003, and sphere assays were grown non-adherently using NeuralBasal medium with Neurocult supplement and growth factors, EGF (20ng/ml), FGF (20ng/mL) and heparin (2mg/mL). Normal human astrocytes were grown in Astrocyte medium (ScienCell cat. #1801) on adherent plants coated with poly-L-lysine (ScienCell cat. #0413).

### Drug treatment and growth assay

Growth assays were conducted by plating 1000 cells/well in 96-well plates with a range of concentrations of TMZ or DSF. All treatments were done in triplicate, and plates incubated at 37°C in a 5% CO2 incubator. After 72 hours, cells are fixed with 2% paraformaldehyde in 100μl PBS, and stained with Hoechst 33342 dye (2 μg/ml) at room temperature for 30 minutes before a wash with 100 μl PBS. Plate analysis and image capture was done using an ArrayScan VTI Reader (Thermal Fisher) [31]. Dimethyl sulfoxide (DMSO) was used to reconstitute TMZ and DSF and was used as a solvent control. Ethanol was used as a control for DEAB.

For analysis of effect of BI-2536 on U251 TMZ resistant cells, U251 cells were treated with 10 uM of TMZ (or DMSO) every 2 days for a total of 7 days. The cells were harvested for protein extraction and immunoblotting was done to examine PLK1 expression. The cells were then re-plated and treated with increasing concentrations of BI-2536 (5-100 nM) for 5 days before the cells were stained with Hoechst dye and quantified on Cellomics. The effect of PLK1 inhibition was investigated using siRNA or BI-2536 as previously described [30].

### Neurosphere Assay

Self-renewal in BT74, GBM4 and SF188 cells was examined using a neurosphere suspension assay (Note: BT74 and GBM4 cells are always maintained as spheres as were the primary isolates described below). Approximately 10 000 cells/well were plated into a low adherent 6 well dish using neurobasal medium supplemented with human recombinant EGF (20 ng/ml), human recombinant FGF (20 ng/ml) and heparin (2 μg/ml) [Stem Cell Technologies]. Primary samples were obtained from adult patients under informed consent according to the BC Cancer Agency guidelines. Tumor cells were isolated as previously described by us [30]. Neurospheres were grown for 5-6 days following plating. Spheres >30 μm were counted and photographed using an Aniovert 40CFL microscope and AxioCam MRc camera. NeuroCult Chemical Dissociation kit (Stem Cell Technologies, cat. #05707) was used to passage cells, which are counted and replated as single cells. All drug treatments of TMZ and DSF were done at the time of plating, and repeated during serial passaging.

### PLK1 regulation and expression

SF188 or U251 cells were treated with DSF (100-500 nM) for 24 hours, proteins were harvested and levels of PLK1 were evaluated by immunoblotting. RNA for gene expression analysis was isolated using Qiagen RNeasy Mini Kit (Cat. #74106). Transcript expression was determined using qRT-PCR with PLK1 Assay on Demand (Applied Biosystems, cat. #4331182). PLK1 expression was silenced using siRNA as previously described [30]. Tumor cell growth following siPLK1 transfection was evaluated compared to scramble control RNA in SF188 cells. PLK1 was also inhibited with BI-2536 and growth was assessed in SF188, BT74, and BT241 all of which are TMZ resistant.

### Immunohistochemisty

Primary adult GBM cases (aBT001 and aBT003) were formalin-fixed, paraffin embedded, sectioned and immunostained for PLK1. PLK1 protein expression was evaluated using LSBio antibody (diluted 1:200, PLK1 rabbit anti-Human polyclonal Antibody LS-B4225- LSBio LifeSpan Bioscience, Seattle, WA). The secondary antibody was universal detection kit from DAKO LSAB2 System-HRP (DAKO, Carpinteria, CA). The MGMT status of these tumors was determined by PCR as previously described [3].

### Statistical Analysis

Experimental data was collected from multiple experiments and reported as the treatment mean ± standard error. Significance was calculated using the Student's t-test, where *p<0.05, and **p<0.01.

## Supplementary Figures


