# Supplementary material for: Disulfiram, a drug widely used to control alcoholism, suppresses self-renewal of glioblastoma and overrides resistance to temozolomide
Source: Oncotarget. 2012 Oct 8;3(10):1112–23. doi: 10.18632/oncotarget.604 (PMC3717961; doi:10.18632/oncotarget.604)
Supplement: Supplementary file 1 [file oncotarget-03-1112-s001.pdf]

Disulfiram, a drug widely used to control alcoholism, suppresses self-renewal of glioblastoma and overrides resistance to temozolomide - Triscott et al

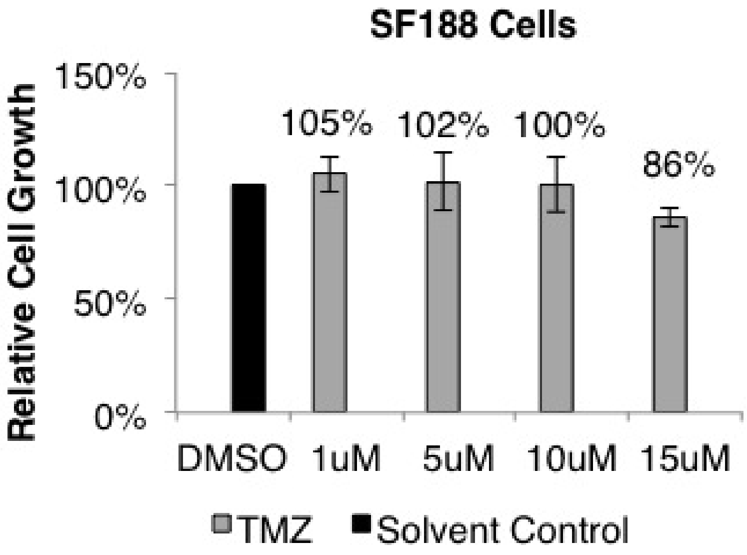

Supplemental Figure 1: SF188 cells are TMZ resistant. SF188 cells plated in triplicate wells of a 72 hrs monolayer growth assays are resistant to TMZ at concentrations of 1, 5, 10 and 15 uM.

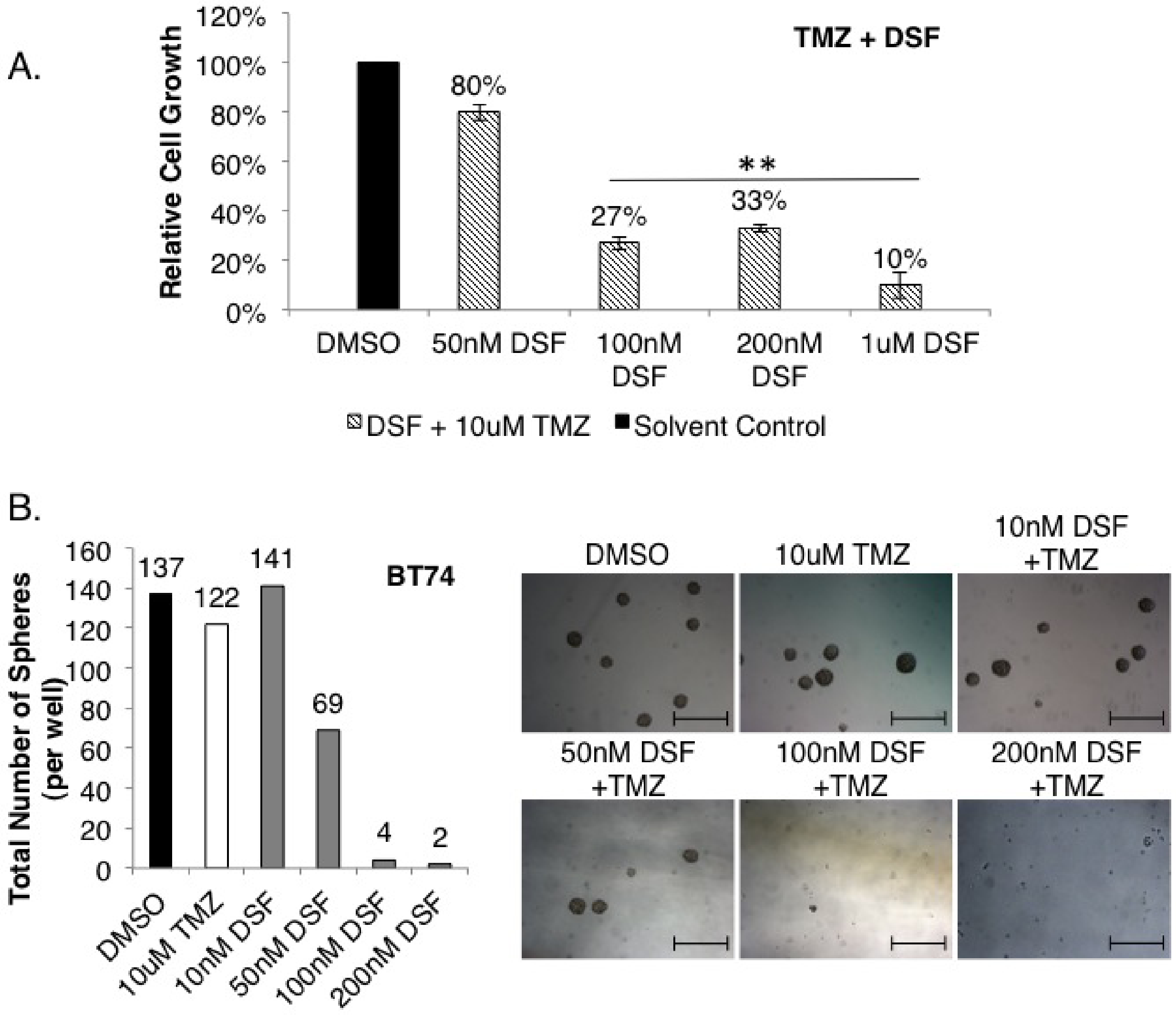

Supplemental Figure 2: Combination treatment of DSF augments TMZ cytotoxicity. (A) SF188 cells treated with DSF (50 nM-1uM), in combination with 10 uM TMZ, in a 72 hrs 96 well plate monolayer growth assay. Cells were plated in triplicate and calculated relative to DMSO control treatment growth [**$p < 0.005$]. (B) BT74 neurosphere assay testing 10 uM TMZ alone, and in combination with DSF (10-200 nM). BT74 spheres >30 uM were counted following 5-6 days of non-adherent growth in neurobasal medium supplemented with growth factors, then chemically dissociated to serial passage and grown for an additional 5-6 days. Morphology of BT74 spheres are shown following 6 days of drug treatment. Scale bar = 200um.

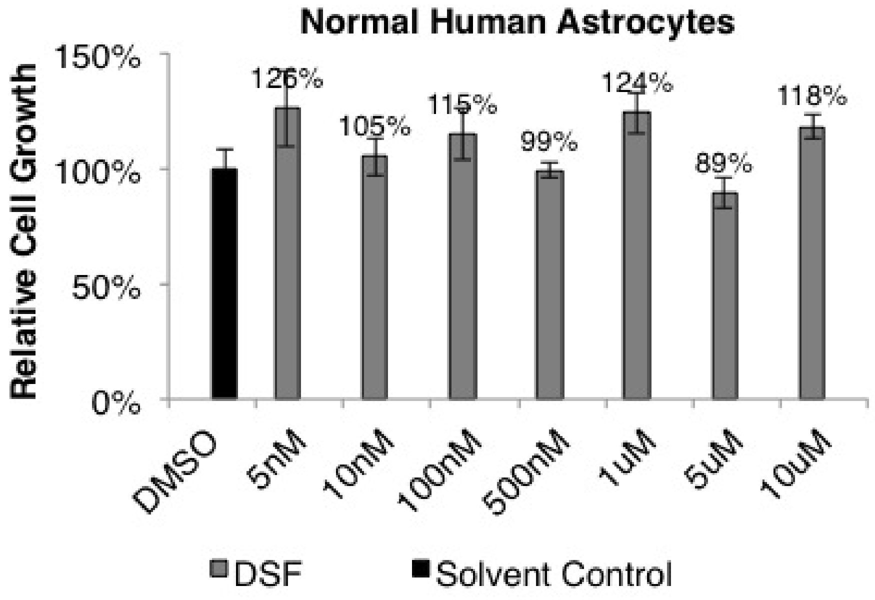

Supplemental Figure 3: High doses of DSF are safe for normal human astrocytes. DSF was given to normal human astrocytes at a concentration range of 5 nM-10 uM and cell proliferation was assessed 72 hrs later. DSF had no effect on the growth of normal cells.

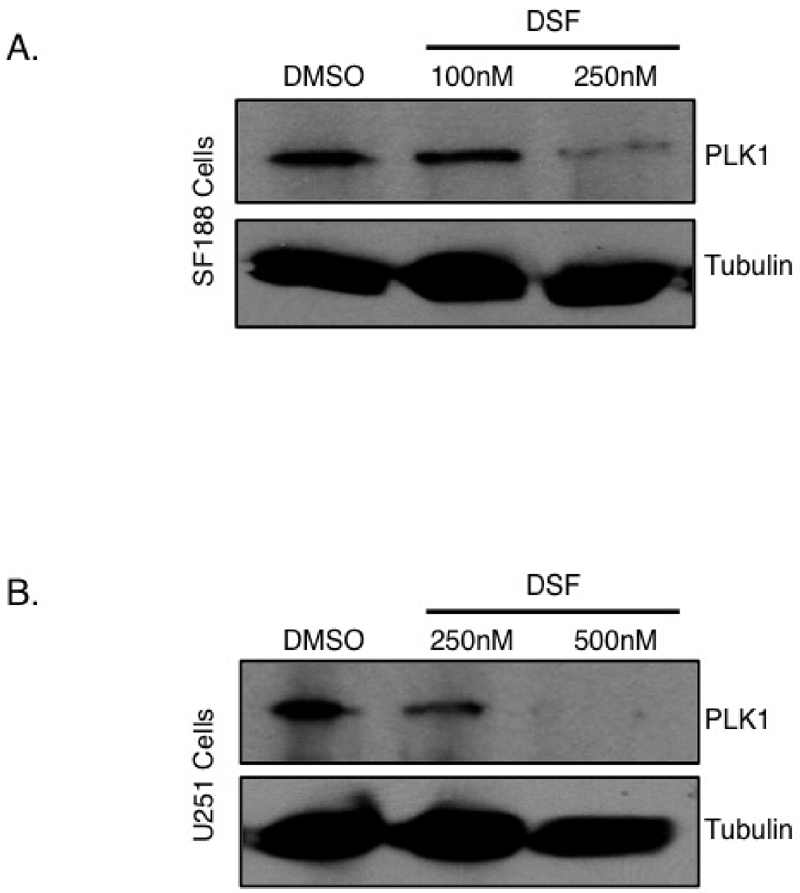

Supplemental Figure 4: DSF inhibits the expression of PLK1. (A) SF188 or (B) U251 cells were treated with DSF and the proteins were harvested 72 hrs later. In both cell lines, 250 nM DSF suppressed PLK1 protein levels based on immunoblotting.
